# Analytical Validation and Clinical Sensitivity of the Belay Summit™ 2.0 Cerebrospinal Fluid Liquid Biopsy Test—An Expanded Comprehensive Genomic Profiling Platform for Central Nervous System Malignancies

**DOI:** 10.3390/cancers18020256

**Published:** 2026-01-14

**Authors:** Sakshi Khurana, Viriya Keo, Alexandra Larson, Vindhya Udhane, Jennifer N. Adams, Anthony Acevedo, Tarin Peltier, Daniel Sanchez, Brett A. Domagala, Samantha A. Vo, Kathleen Mitchell, Dean Ellis, Baymuhammet Muhammedov, Samer I. Al-Saffar, Kyle M. Hernandez, Chetan Bettegowda, Christopher Douville, Kala F. Schilter, Qian Nie, Honey V. Reddi

**Affiliations:** 1 Belay Diagnostics, Suite 530, 1375 W. Fulton St., Chicago, IL 60607, USA; 2 Johns Hopkins University School of Medicine, Baltimore, MD 21205, USA

**Keywords:** CSF liquid biopsy, comprehensive genomic profiling, CNS tumors

## Abstract

Background/Objectives: The latest National Comprehensive Cancer Network (NCCN) Central Nervous System (CNS) Guidelines recommend utilizing next-generation sequencing (NGS) to enable comprehensive genomic profiling (CGP) as the preferred approach for molecular characterization of central nervous system (CNS) malignancies. CNS malignancies present distinct challenges due to the infeasibility of tissue-based testing for many patients and the restrictive nature of the blood–brain barrier (BBB) making plasma-based liquid biopsy an ineffective alternative. Recent advances in liquid biopsy have extended molecular testing beyond plasma to include cerebrospinal fluid (CSF), which serves as a valuable source for tumor-derived nucleic acids. Methods: The Belay Summit™ 2.0 is a high-throughput CGP assay capable of detecting multiple variant types, including single nucleotide variants (SNVs) and small insertion and deletions (Indels), copy number variations (CNVs), gene fusions, splice variants, and immunotherapy biomarkers such as microsatellite instability (MSI) and tumor mutational burden (TMB). This study details the analytical and clinical validation of Summit™ 2.0 to assess its technical performance and clinical sensitivity. Analytical validation was conducted using 68 specimens, demonstrating robust and reproducible detection of all variant types with 15 ng of CSF-derived total nucleic acid (tNA). Results: The analytical sensitivity of the Belay Summit™ 2.0 assay for SNVs and Indels was determined to be 96.7% with a 100% limit of detection (LoD) at a variant allele frequency of 0.3%. Clinical validity was evaluated across a cohort of 118 CSF specimens, including both primary and metastatic CNS tumors, demonstrating 96% sensitivity and 98% specificity. Conclusions: These findings support the use of the Belay Summit™ 2.0 assay for accurate and reproducible genomic profiling of CNS tumors using tumor-derived nucleic acids from CSF in patients for whom tissue-based testing is considered infeasible, unsafe, or not deemed by the prescribing physician to be clinically appropriate.

## Simple Summary

Detection of cancers of the central nervous system (CNS) including the brain and spine using blood has several limitations due to the presence of the blood brain barrier. Using cerebrospinal fluid (CSF) as a minimally invasive method to inform diagnosis of CNS cancers has been clearly demonstrated. This study documents the validation results of the Belay Summit™ 2.0 test using CSF which demonstrated high sensitivity (true cancer cases) and specificity (non-cancer cases) in the detection of CNS cancers.

## 1. Introduction

Comprehensive genomic profiling (CGP) has become an essential tool for guiding diagnosis, prognosis, targeted therapy selection, and clinical trial enrollment for primary and metastatic solid tumors [1]. Effective profiling requires detection of clinically significant variant types including single-nucleotide variants (SNVs), copy number variations (CNVs), fusions, and immunotherapy biomarkers such as microsatellite instability (MSI) and tumor mutational burden (TMB). MSI and high TMB predict the response to immune checkpoint blockade therapy, driving FDA approvals of tumor-agnostic treatments such as pembrolizumab [2]. Driven by rising cancer prevalence and a shift towards minimally invasive diagnostic strategies as an alternative when tissue-based testing is infeasible or unsafe, the liquid biopsy market in the United States is experiencing rapid growth [3]. While circulating tumor DNA (ctDNA) from plasma or serum enables therapy selection and disease monitoring in many cancers, genomic profiling of central nervous system (CNS) malignancies utilizing liquid biopsy presents unique challenges due to the blood–brain barrier, which limits tumor-derived nucleic acids in circulation [4,5]. Cerebrospinal fluid (CSF) provides a direct window into the CNS tumor microenvironment, capturing DNA shed from both primary and metastatic lesions and addressing the limitations of traditional diagnostics such as MRI, tissue biopsy, and CSF cytology [6]. The advances in CSF tumor-derived DNA (CSF-tDNA) testing in recent years has enabled its inclusion into the NCCN guidelines for both primary and metastatic brain cancers.

**Figure 1:**
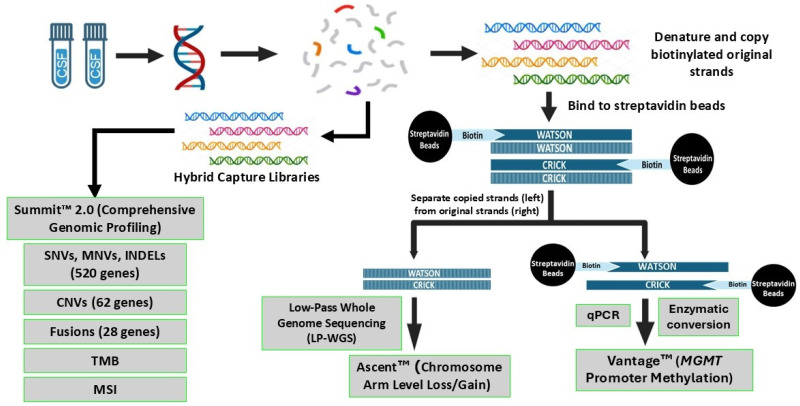
Summit™ 2.0 workflow including the Chromosomal Arm-Level Aneuploidy [7] and the Belay Vantage assay [13] which have been previously validated. Summit™ 2.0 content showing the different variant types and biomarkers evaluated. SNVs, single nucleotide variants; MNVs, multi-nucleotide variants; Indels, insertions/deletions; CNVs, copy-number variants; TMB, tumor mutational burden; MSI, microsatellite instability.

To better inform the treatment and management of CNS malignancies when tissue-based testing is infeasible or unsafe, CSF-based NGS assays such as Belay’s Summit™ test [7] have been developed to enable sensitive, high-throughput characterization of CNS tumors, offering a minimally invasive alternative to tissue biopsy while expanding the clinical utility of liquid biopsy in neuro-oncology [8,9,10,11]. Summit™ 1.0 [7] combined targeted evaluation of 32 genes (single nucleotide variants—SNVs and insertion/deletions—indels) and low-pass whole-genome sequencing (LP-WGS) for aneuploidy using the MethySaferSeqS [12] duplex sequencing methodology for high-sensitivity detection (~0.2% variant allele frequency (VAF)). Summit™ 2.0 builds on this first-generation test, expanding to evaluate 520 genes for SNV and Indel detection along with additional biomarkers of clinical relevance, including fusions, gene-level copy-number variants (CNVs), microsatellite instability (MSI), and tumor mutational burden (TMB) (Figure 1). Summit™ 2.0 was developed similarly to 1.0 in that it facilitates the ability to assess genome-wide aneuploidy through LP-WGS [7] and the simultaneous evaluation of *MGMT* promoter methylation status using the Belay’s Vantage™ test [13] with a single CSF input (Figure 1). This study describes the analytical and clinical validation of the Belay Summit™ 2.0 expanded panel component for biomarker detection, using tumor-derived total nucleic acid (tNA) from patients with primary and metastatic CNS tumors.

## 2. Materials and Methods

### 2.1. Belay Summit™ 2.0 Content

The Summit™ 2.0 CGP next-generation sequencing (NGS) test investigates tNA extracted from CSF for clinically relevant biomarkers. The methodology involves evaluation of 520 genes for SNVs/MNVs and Indels, 62 genes for CNVs, and 28 genes for fusions (Supplementary Table S1) (Figure 1). The larger panel is required to be able to report on biomarkers such as MSI and particularly TMB, which requires at least 1–2 Mb of real data [14]. Summit™ 2.0 enables the evaluation of most genes included in the World Health Organization (WHO) classifications [15] and National Comprehensive Cancer Network (NCCN) guidelines for both adult [16] and pediatric [17] CNS tumors.

### 2.2. Sample Cohort

A total of 186 specimens were evaluated in validation studies (Table 1). The size of the sample cohorts for each study was based on professional society guidelines for the validation of oncology panels [18,19,20].

The analytical validation cohort of 68 samples included 20 no-template controls (NTCs), 11 contrived controls, and 37 clinical CSF specimens presumed to be non-cancerous or normal (*n* = 20) or from individuals with a known diagnosis or suspicion of cancer (*n* = 17). Contrived controls included Horizon Mimix™ Tru-Q1 (5% Tier) (Horizon, Cambridge, MA, USA, Cat# HD728), Horizon Mimix™ Tru-Q0 (100% Wild-type) (Horizon, Cambridge, MA, USA, Cat# HD752), Horizon Mimix™ Structural Multiplex gDNA Reference Standard (Horizon, Cambridge, MA, USA, Cat# HD753), Horizon Mimix™ OncoSpan cfDNA Reference Standard (Horizon, Cambridge, MA, USA, Cat# HD833), Seraseq^®^ ctDNA Complete™ Mutation Mix WT (0%) (Milford, MA, USA, Cat# 0710-0533), Seraseq^®^ ctDNA Complete™ Mutation Mix AF5% (Milford, MA, USA, Cat# 0710-0528), Seraseq^®^ Solid Tumor CNV Mix +6 copies (Milford, MA, USA, Cat #0710-2867), Seraseq^®^ gDNA MSI-High Mix (Milford, MA, USA, Cat# 0710-1670) and MSI Reference Panel Mix AF5% (Milford, MA, USA Cat# 0710-1675), and Seraseq^®^ gDNA TMB Reference Panel Mix (Milford, MA, USA, Cat# 0710-2463).

Clinical validation studies evaluated 118 clinical CSF samples with a known diagnosis or suspicion of cancer (*n* = 78) and no known cancer present (*n* = 40). Demographics for the cohort are described in Table 2. De-identified CSF samples without any clinical information from individuals presumed not to have cancer (remnant samples of individuals that were received for testing of non-cancer indications, such as Lyme disease, HSV infection, etc.) were commercially purchased (LabCorp, Burlington, NC, USA). CSF specimens that were tissue biopsy-matched or had a definitive diagnosis from individuals with a non-cancerous neurological condition after treatment for cancer but no active disease, a known diagnosis or suspicion of cancer, and limited demographic information (age range, sex assigned at birth, and ethnicity) were obtained from Johns Hopkins University (JHU) under an institutional IRB (IRB00420181) in compliance with the principles of the Declaration of Helsinki and the Health Insurance Portability and Accountability Act (HIPAA).

### 2.3. Summit™ 2.0 Methodology—Sequencing and Data Analysis

A total of 1–25 ng of sheared tNA extracted from CSF was used to index libraries prior to next-generation sequencing [7]. Normalized library pools were loaded onto a 10 B flow cell (Illumina Inc., San Diego, CA, USA) for 2 × 151 bp sequencing on a Novaseq X Plus (Illumina Inc.). Variants (SNVs, Indels, CNV, and Fusions) and biomarkers (TMB and MSI) were called against the human genome build reference hg19 using the Summit™ Omics pipeline (version 1.0.0) developed at Belay Diagnostics (Chicago, IL, USA).

Raw reads from sequencing were filtered with the allele counts (ACs), total depth, quality of reads, base quality [Phred score of ≥30 (Q30)], and VAFs before evaluation. Data are reported for all variants detected in the raw data that passed established thresholds, or as reportable variants (clinically significant variants [21]), in the context of thresholds established for VAFs and ACs. All cutoffs were evaluated to ensure a sensitivity ≥ 90% and specificity ≥ 95% in accuracy studies.

## 3. Results

### 3.1. Analytical Validation of Summit™ 2.0

The analytical validation (AV) of Summit™ 2.0 evaluated the limit of blank (LoB), limit of input (LoI), limit of detection (LoD), accuracy (sensitivity/positive percentage agreement (PPA) and specificity, negative percentage agreement (NPA), and precision (repeatability and reproducibility). Quality metrics (Table 3) for the Summit™ 2.0 expanded panel from library preparation to run level and sample level were established in previously published methods [7], vendor/manufacturer instructions, and initial feasibility studies. The average raw depth coverage of all AV samples was 52,940×, which is significantly higher than the recommended raw depth coverage of CSF samples at 10,000–20,000× [22].

### 3.2. Summit™ 2.0 Demonstrated an Exceptionally Low Overall False Positive Rate of 0.02% in LoB Studies

No-template controls (NTCs), wild-type (WT) contrived controls, and 20 non-cancer CSF specimens were used to determine the highest false positive measurement results for blank samples. A stated probability α (type I error rate) of a 5% false positive (FP) rate was applied [23]. When sequenced, the NTCs generated, on average, excluding when the reads that were generated were too few to be captured, 182 million reads, only 0.5% of which aligned to the genome, in contrast to the contrived controls and CSF specimens, which averaged 811 million reads and a 95.3% alignment rate.

Variants that met all quality filtering criteria (as outlined in Materials and Methods) were included in the final analysis. No variants were detected in the NTC samples. Likewise, no variants known to be present only in the mutation-positive contrived controls were observed in the wild-type controls, demonstrating a 0% false positive (FP) rate. Across all presumed normal CSF samples, approximately 25,000 variants were identified: of these, only 4 met reporting thresholds for classification as clinically significant variants, corresponding to an overall FP rate of 0.02%.

### 3.3. Limit of Input for Summit™ 2.0 Was Demonstrated to Be ≥15 ng for All Biomarkers

**Figure 2:**
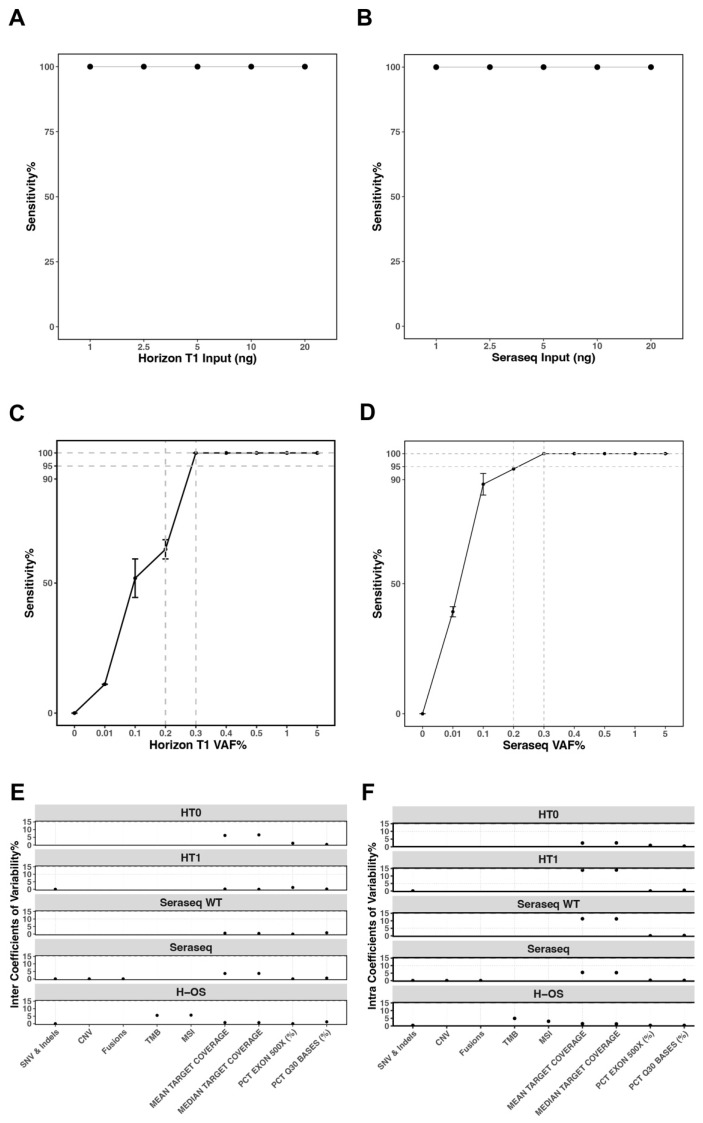
(**A**,**B**) Limit of input: Contrived controls (Horizon T1 and Seraseq) evaluated at different inputs at a variant allele frequency (VAF) of 5% demonstrated 100% sensitivity by detecting all 26 variants. (**C**,**D**) Limit of detection: Contrived controls (Horizon T1 and Seraseq) evaluated at 5 ng into the library at VAFs of 0–5% with 100% sensitivity at 0.3% VAF. (**E**,**F**) Repeatability and reproducibility: Using five contrived controls (Horizon T0, T1, OncoSpan, and Seraseq wild-type and mutant), intra-assay repeatability (same samples run in duplicate by one operator on the same day) (**E**) and inter-assay reproducibility (same samples run in duplicate by different operators on different days), (**F**) were 100%. All quality metrics showed a coefficient of variability <15%. HT0, Horizon T0; HT1, Horizon T1; WT, wild-type; H-OS, Horizon-OncoSpan; SNV, single nucleotide variant; Indel, insertion/deletion; CNV, copy-number variant; TMB, tumor mutational burden; MSI, microsatellite instability; PCT, percent.

To establish the lowest input for detecting different variant types, all variants including SNV/Indels, CNVs, Fusions, TMB, and MSI were evaluated at different tNA input levels. **SNVs/INDELs**: The Horizon T1 and Seraseq Mutation Mix (non-sheared) contrived controls were evaluated at 1, 2.5, 5, and 10 ng inputs during both shearing and library preparation to establish the lowest input level that consistently enabled detection of SNVs and MNVs at a 5% VAF. All expected true positive (TP) variants, 9 from the Horizon T1 control and 17 from the Seraseq Mutation Mix, were successfully detected in all three replicates at the 1 ng shearing input, confirming this to be the limit of input for SNVs and Indels (Figure 2A,B). **CNVs:** The Horizon Structural Multiplex, Seraseq CNV, Seraseq Mutational Mix contrived controls were analyzed at input levels of 2.5, 5, and 10 ng. All CNVs known to be present were successfully identified at the lowest input of 2.5 ng. Gene-level amplification was defined by a fold change threshold of ≥2, which was consistently detected with all inputs (Table 4). **Fusions**: The Horizon Structural Multiplex and Seraseq Mutational Mix controls were evaluated at input levels of 2.5, 5, and 10 ng. All expected fusions were successfully detected across all input levels for both controls. Detection thresholds were defined as fusions supported by at least two sequencing reads, with at least one breakpoint located within 1 of the 28 target genes. Based on these results, the LoI for reliable fusion detection was established at 2.5 ng (Table 4). Of note, in contrast to other gene fusions which are called structural breakpoint events, *EGFRvIII* (*EGFR*; chromosome 7:55,086,714–55,324,313) and *MET* exon 14 skipping (*MET*; chromosome 7:116,312,444–116,438,440) were inferred by copy-number and splicing variants, respectively. **MSI**: The Seraseq MSI Normal and MSI High in the Reference Panel Mix with known MSI status were evaluated to determine the minimum input required for accurate MSI-High (MSI-H) calling across four input ranges, <5 ng, 5–7.5 ng, 7.5–10 ng, and >10 ng into the library. All MSI-H samples exhibited more than 5% unstable microsatellite sites, establishing 5% as the threshold for MSI-H classification. Using this criterion, MSI-H status was consistently detectable with a library input of 5 ng or higher (Table 4). **TMB**: The LoI of TMB was also evaluated using the TMB Reference Panel Mix and Horizon OncoSpan controls across the same input ranges as MSI. TMB-high status was defined as a total TMB score greater than 10, which was reliably detected at input levels above 7.5 ng into the library (Table 4).

### 3.4. Limit of Detection for Summit™ 2.0 for SNVs and Indels Is 0.3% at LoD100

To determine the lowest VAF that can be consistently detected, two contrived controls, Horizon T1 and Seraseq Mutation Mix, were evaluated, one mimicking sheared tNA (Horizon) and the other mimicking unsheared tNA (Seraseq). Both controls were evaluated at VAF levels of 0%, 0.01%, 0.1%, 0.2%, 0.3%, 0.4%, 0.5%, 1%, and 5%, with three replicates per VAF at a 10 ng input into shearing (Horizon) or 5 ng into the library (Seraseq). Of a total of 26 variants that were analyzed for all VAFs, both controls consistently detected all variants at a 0.3% VAF, demonstrating 100% sensitivity and establishing this to be the LoD for SNVs and Indels (Figure 2C,D). Additionally, the unsheared Seraseq control demonstrated 95% sensitivity at a VAF of 0.2% (Figure 2D).

### 3.5. Summit™ 2.0 Analytical Precision Was Demonstrated to Be 100%

Five contrived controls across multiple biomarkers were processed in replicates (Horizon and Seraseq: wild-type and mutant, and Horizon OncoSpan) to evaluate intra-assay repeatability (same samples run in duplicate on same plate by one operator on the same day) and inter-assay reproducibility (same samples run in duplicate by different operators on different days). Results showed repeatability and reproducibility to be 100% with all quality metrics having a coefficient of variability less than 15% [24] (Figure 2E,F).

### 3.6. Analytical Accuracy of Summit™ 2.0 Was >96%

Analytical accuracy of Summit™ 2.0 was evaluated using 44 specimens, including 37 clinical CSF specimens with known cancer (*n* = 17) and true negative specimens (*n* = 20) and 7 blinded contrived controls. Detected variants were compared with the known variants of each sample to determine analytical accuracy (Table 5). SNVs/Indels: Of the 31 variants detected by Summit™ 1.0, 27 variants (true positive, TP) were also identified by Summit™ 2.0. Among the four missing variants (false negative, FN), two were present in the raw VCF file and one was undetectable due to genomic location, resulting in an analytical sensitivity and positive percent agreement (PPA) of 96.43%. Six variants were uniquely detected by Summit™ 2.0 (false positives, FPs), of which five were not covered by Summit™ 1.0, yielding an analytical specificity of 96.43%. **CNVs**: In addition to the 15 CNVs detected in the three contrived controls evaluated, another 10 CNVs were detected in both Summit™1.0 and 2.0 cancer specimens (*n* = 5), which suggests 100% analytical sensitivity/PPA and specificity, respectively. **Fusions**: A total of 19 unique fusions were detected in both the CSF cancer specimens and 3 contrived controls, suggesting 100% analytical sensitivity/PPA and specificity, respectively. **TMB and MSI**: TMB (not detected, low or high) and MSI (MSS or MSI-High) status was called as expected (based on known status) in all samples evaluated, demonstrating 100% analytical sensitivity/PPA and 100% specificity.

### 3.7. Clinical Sensitivity and Specificity of Summit™ 2.0

A total of 118 clinical CSF specimens from individuals with varying demographics and with a broad range of tNA inputs were analyzed to assess the clinical sensitivity and specificity of the Summit™ 2.0 assay to inform cancer versus non-cancer status. These specimens included 40 true negative (TN) samples and 78 true positive (TP) samples (Table 6).

All 118 samples successfully completed the Summit™ 2.0 workflow, demonstrating consistent performance across variable input ranges for SNVs/Indels, CNVs, and Fusions. Of the 118 samples, 10 failed for both TMB and MSI (8.5%) when the average input into the library was <4 ng, with an additional 39 of the remaining 108 (36%) failing for only TMB when the average input was <6 ng. These results confirm the AV studies that >10–15 ng is required for reportability of both these biomarkers.

**Figure 3:**
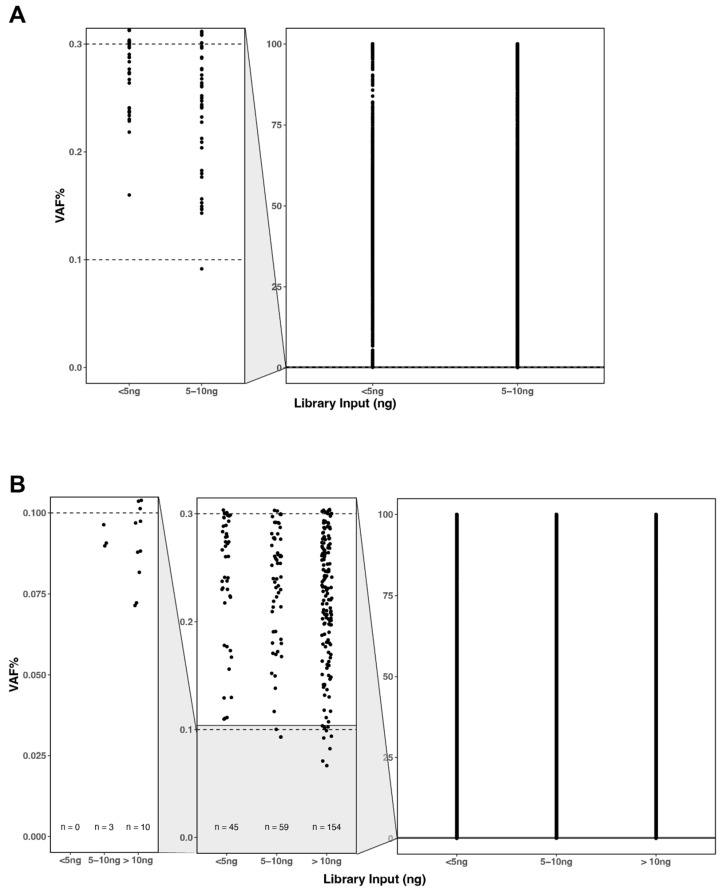
Threshold of positivity in (**A**) analytical validation and (**B**) clinical validation grouped by input into the library. Variant allele frequencies (VAFs) > 0.3% were detected in all input groups but variants with VAF < 0.3% were not detected in inputs < 5 ng for both validation cohorts.

A total of 159,136 variants were identified in the cohort, spanning a wide spectrum of VAFs from 0.06% up to 100%, reflecting the assay’s ability to detect both low-level and high-level VAFs. Consistent with the AV results (Figure 3A), variants with VAFs ≥ 0.3% were reliably detected across all input levels, independent of the amount of starting tNA available. The detection of lower-frequency variants (<0.3% VAF) improved as input increased, with samples prepared from more than 10 ng of tNA showing the largest number of variants below this threshold, consistent with greater molecular diversity and deeper unique coverage at higher inputs. No variants with VAFs below 0.1% were identified in samples with library inputs less than 5 ng (Figure 3B), which also reflects the findings from AV (Figure 3A).

**Figure 4:**
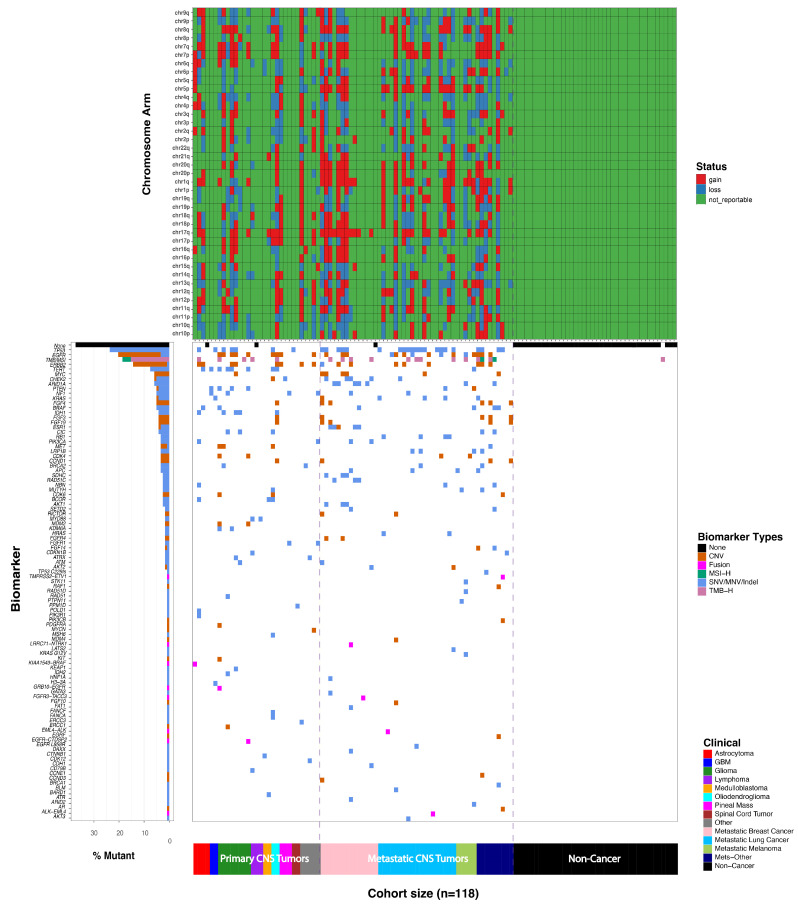
Clinically significant variants detected in the clinical validation (CV) cohort. Of the 118 samples, clinically significant single nucleotide variants (SNVs), insertion/deletions (Indels), multi-nucleotide variants (MNVs), copy-number variants (CNVs), fusions and/or tumor mutational burden (TMB), and microsatellite instability (MSI) (bottom panel) were detected in 75 of 78 true positive cases, with aneuploidy (top panel) being detected in 47 of the 75 positive cases (63%). No clinically significant variants were detected in 39 of the 40 true negative samples; a single false positive was reported due to high-TMB. CNS, central nervous system; GBM, glioblastoma; Mets, metastatic.

Among the 78 true positive cases, Summit™ 2.0 correctly identified 75 cancer samples with a broad range of clinically reportable alterations across multiple variant classes (Table 6). Sixty-six of the seventy-five positive samples (88%) harbored at least one reportable SNV/MNV/Indel, with VAFs spanning from 0.3% to 100%. Thirty-four of the seventy-five cases (45%) carried reportable CNV events, and eight (13%) samples had reportable fusion events detectable. Fourteen (19%) samples had high TMB status, eleven of which were metastatic CNS tumors. No samples with MSI-H status were detected in the CV cohort. Arm-level aneuploidy (as validated in Summit™ 1.0) was observed at high prevalence, with 47 of the 75 (63%) samples having more than three-chromosomal arm-level loss and/or gain (Figure 4). No clinically significant variants were detected in three cancer specimens, a pilocytic astrocytoma, a lymphoma, and a metastatic breast cancer, resulting in a false negative rate of 3.8% (3 of 78).

The true negative specimens (*n* = 40) included the following conditions, 11 trigeminal neuralgia, 6 Chiari malformations, 6 hydrocephalous, 13 non-neoplastic, and 1 each of the following: hemifacial spasm, benign neurology, epidermoid cyst, and spinal root enhancement. One sample was called positive due to having high-TMB, resulting in a false positive rate of 2.5%, suggesting that high-TMB by itself cannot be used as a biomarker to assign cancer in the absence of other clinically significant variants [25,26]. Overall, Summit™ 2.0 achieved a clinical sensitivity (positive percent agreement, PPA) of 96% and a clinical specificity (negative percent agreement, NPA) of 98% (Table 6), highlighting the robustness of the test to be used for CSF liquid biopsy.

### 3.8. Summit™ 2.0 Demonstrates Increased Detection Yield Compared to Summit™ 1.0

Post validation and upon clinician request, 10 patient samples that were negative in Summit™ 1.0 were retested using Summit™ 2.0. Clinically significant variants were detected in 8 of the 10 samples, demonstrating an 80% increased detection yield. Reported variants detected included SNVs/Indels in the *FANCD2*, *NF1*, *CHECK2*, *PPM1D*, and *ARID1A* genes, along with *MDM2* amplification, and gene fusions of *EML4-ALK* and *KIAA1549-BRAF* (Table 7). Two samples, a breast carcinoma and a germ cell tumor, remained negative with Summit™ 2.0.

Twenty-four cases processed for Summit™ 2.0 that had accompanying tumor genomic profiling results (Table 8) were evaluated to identify complete or partial concordance in terms of reportable variant detection. Four of the twenty-four cases (17%) were negative for Summit™ 2.0 compared to tumor results. Of the remaining 20 Summit 2.0 positive cases, the concordance of Summit was observed in 17 cases (85%), demonstrating the clinical utility of CSF as a source to inform detection of CNS tumors.

## 4. Discussion

Traditional diagnostic and management approaches for CNS tumors such as MRIs, CSF cytology, and tissue biopsies have several limitations driving the need for minimally invasive liquid biopsy tests. Additionally, the World Health Organization (WHO) [15] and the National Comprehensive Cancer Network (NCCN) guidelines [16,17] have shifted toward integrated diagnostic approaches which now include molecular characterization and CSF-tDNA testing. To address limitations of current diagnostic methods including the much lower sensitivity of plasma for the detection of CNS tumors [27,28], Summit™ 1.0 [7], a 32-gene panel (SNVs/Indels) with low pass WGS (chromosomal arm-level loss and/or gain) was developed as a first-generation CSF liquid biopsy test, with a demonstrated clinical sensitivity of 90% and specificity of 95% [7]. However, the content was not adequate to cover all guideline requirements for both adult and pediatric CNS malignancies. To address these gaps and to improve sensitivity, Summit™ 2.0 was developed as a CGP assay that included an expanded panel of 520 genes for SNVs/Indels, 62 genes for CNVs, 28 genes for fusions, and additional biomarkers such as TMB and MSI. This study documents the analytical validation of Summit 2.0 with a clinical sensitivity of 96% with a specificity of 98%, much higher than other tests commercially available tests for CSF liquid biopsy testing, whose values range from 40 to 80% [22,29,30]. While studies to demonstrate the clinical utility of Summit™ 2.0 are ongoing, the AV/CV performance of this test highlights key areas of clinical impact due to its higher sensitivity and specificity.

### 4.1. Higher Detection Yield

Analytical sensitivity of Summit™ 2.0 for SNVs and Indels was determined to be 96.7%, with a 100% limit of detection at a 0.3% VAF, a clinical sensitivity of 96%, and a specificity of 98%, demonstrating superior performance compared to Summit™ 1.0, which had a clinical sensitivity of 90% and specificity of 95% [7]. The retesting of 10 samples that were negative for Summit™ 1.0 detected a reportable variant in 8 cases for an improved detection rate of 80% with Summit™ 2.0.

### 4.2. Increased Sensitivity of CSF Liquid Biopsy to Inform on CNS Tumors

Evidence clearly demonstrates the clinical utility of CSF liquid biopsy compared to plasma for the evaluation of CNS malignancies particularly because of the blood–brain barrier [31]. Since its launch, the clinical impact of Summit™ 1.0 as a CSF liquid biopsy test has been demonstrated in multiple case studies to obviate biopsy [10], track therapy response [9,32], and inform diagnosis and management of leptomeningeal disease [11,32]. Evaluating 24 cases assessed with Summit™ 2.0 that had prior tumor genomic profiling results showed a concordance of 90% of Summit™ 2.0 with tumor results, demonstrating its clinical utility as a source to inform on the detection of CNS malignancies. Prospective studies are required to corroborate these observations.

### 4.3. Enhanced Therapeutic Decision Making

The ability to detect tumor agnostic biomarkers such as *NTRK* fusions, TMB, and MSI allows for additional treatment options to become available to patients, such as NTRK inhibitors—entrectinib and larotrectinib—to treat solid tumors in adults and children and immune checkpoint inhibitors such as pembrolizumab and nivolumab, options that would not be recommended based on SNV status alone. Summit™ 2.0 thus facilitates therapeutic decision making with its expanded biomarker content.

## 5. Conclusions

In conclusion, Summit™ 2.0 is a highly sensitive and specific comprehensive genomic profiling test that can evaluate a variety of gene variants (SNVs/Indels/CNVs, gene fusions, and splice variants), as well as TMB and MSI with 10–15 ng of tNA to inform treatment and management of primary and metastatic CNS tumors as a liquid biopsy option when tissue-based testing is infeasible or unsafe.

## Figures and Tables

**Table 1:** Specimens used for validation studies (*n* = 186).

Specimen Type	Study
No-template control (*n* = 20)	AV: LoB
Contrived controls (*n* = 11)	AV: LoI, LoD, precision
Clinical CSF, known cancer samples (*n* = 17)	AV: accuracy
Clinical CSF, presumed normal specimens (*n* = 20)	AV: LoB, accuracy
CSF cancer samples (true negatives) (*n* = 40)	CV: clinical sensitivity
CSF cancer samples (true positives) (*n* = 78)	CV: clinical sensitivity

AV, analytical validation; CSF, cerebrospinal fluid; LoB, limit of blank; LoD, limit of detection; LoI, limit of input.

**Table 2:** Demographic characteristics of the clinical validation cohort.

Individual Cases (*n* = 118)	Non-Cancer (*n* = 40; 34%)	Cancer (*n* = 78; 66%)
**Sex assigned at birth**		
Male	17 (42.5%)	31 (39.7%)
Female	23 (57.5%)	40 (51.3%)
Not Available	0 (0%)	7 (9%)
**Race**		
White	27 (67.5%)	28 (35.9%)
Black or African American	4 (10%)	4 (5.1%)
Other	5 (12.5%)	12 (15.4%)
Unknown	4 (10%)	34 (43.6%)
**Age range (years)**		
0–18	9 (22.5%)	3 (3.8%)
19–39	10 (25%)	12 (15.4%)
40–65	15 (37.5%)	36 (46.2%)
>65	6 (15%)	17 (21.8%)
Not Available	0 (0%)	10 (12.8%)

Other in the “Race” cohort includes Asian, Pacific Islander, Middle Eastern, and Hispanic/Latino.

**Table 3:** Quality threshold metrics for Summit™ 2.0.

Sample Type	Analytical Validation (Range)	Clinical Validation (Range)
Shearing Input (ng)	1–40	10–25
Library Input (ng)	1–20	1–25
Passing Filter Rate	74.1–86.5	76.8–86.63
Percentage of Q30	91.0–94.9	92.91–94.83
Total PF Reads	1.16 × 10^8^–1.46 × 10^9^	2.50 × 10^8^–1.04 × 10^9^
Mean Target Coverage	104–3364	196–2608

**Table 4:** Limit of input.

	SNV/INDEL	CNV	Fusion		MSI		TMB	
Sample Size (*n*)	2	3	2	Sample Size (*n*)	MSS (1)	MSI-H (1)	TMB-L (1)	TMB-H (2)
Input DNA (ng)	Expected Calls: 26	Expected Calls: 15	Expected Calls: 6	Input DNA (ng)	Unstable Sites% < 5	Unstable Sites% ≥ 5	Total TMB < 10	Total TMB ≥ 10
1	26	NA	NA	<5	NA	NA	NA	NA
2.5	26	15	6	5–7.5	NA	NA	NA	NA
5	26	15	6	7.5–10	1	1	1	2
10	26	15	6	>10	1	1	1	2

SNV, single nucleotide variant; INDEL, insertion/deletion; CNV, copy-number variant; MSI, microsatellite instability; TMB, tumor mutational burden; MSS, microsatellite stable; MSI-H, microsatellite instability high; TMB-L, tumor mutational burden low; TMB-H, tumor mutational burden high.

**Table 5:** Accuracy of Summit™ 2.0.

		Number of Variants Called					
Variants/Biomarkers Evaluated	Number of Samples Evaluated (*n* = 44)	True Positive	False Positive	True Negative	False Negative	Analytical Sensitivity (PPA)	Analytical Specificity
SNV/INDEL	17	27	6	25,858	4	96.43%	96.43%
CNV	8	13	0	133	0	100%	100%
Fusion	6	19	0	22	0	100%	100%
TMB	2	1	0	1	0	100%	100%
MSI	4	1	0	3	0	100%	100%

SNV, single nucleotide variant; INDEL, insertion/deletion; CNV, copy-number variant; MSI, microsatellite instability; TMB, tumor mutational burden.

**Table 6:** Clinical sensitivity of Summit™ 2.0 to detect cancer in primary and metastatic central nervous system (CNS) tumors (*n* = 118).

Cancer Type	Cases, *n*	Sensitivity (%)
True Negatives (non-cancer)	40	2.5 (FP)
True Positives (cancer)	78	96 (TP)
**Primary CNS cancer (*n* = 30) [sensitivity of 92%]**		
Glioma	8	100
Astrocytoma	4	75
Lymphoma	3	67
Pineal Mass	3	100
Glioblastoma	2	100
Medulloblastoma	2	100
Oligodendroglioma	2	100
Spinal Cord Tumor	2	100
Choroid Plexus Papilloma	1	100
Germ Cell Tumor	1	100
Myelodysplastic Syndrome	1	100
Rhabdosarcoma	1	100
**Metastatic CNS cancer (*n* = 48) [sensitivity of 98%]**		
Lung	19	100
Breast	14	93
Skin	5	100
Kidney	2	100
Colon	3	100
Gastric	2	100
Prostate	1	100
Unknown Primary	2	100

**Table 7:** Improved detection yield of Summit™ 2.0 in Summit™ 1.0 negative cases (clinically significant variants reported based on guidelines) [15,16,21].

Tumor	Summit™ 2.0	SNV/INDEL	CNV	Fusion	Aneuploidy
Glioma	Positive	ARID1A W2050 *, *FANCD2* c.1134+2T>C	None	*KIAA1549::BRAF*	1p/19q Codeletion
Astrocytoma	Positive	*FANCD2* c.990-1G>A, NF1 Q1723 *	None	None	None
Germ Cell Tumor	Negative	None	None	None	None
Chronic Lymphocytic Leukemia	Positive	CHEK2 E394K	None	None	None
Lung Adenocarcinoma	Positive	None	MDM2 Amplification	*EML4::ALK*	High (>5 events)
Lung Adenocarcinoma	Positive	None	None	*EML4::ALK*	None
Breast Carcinoma	Positive	PPM1D R552 *	None	None	None
Breast Carcinoma	Negative	None	None	None	None
Melanoma	Positive	CHEK2 E64K	None	None	None
Soft Tissue Sarcoma	Positive	NF1 L2711fs	None	None	None

SNV, single nucleotide variant; INDEL, insertion/deletion; CNV, copy-number variant; *, stop-gain variant.

**Table 8:** Concordance of Summit™ 2.0 with previous tumor genomic profiling results.

ID	Tissue of Origin	Variants Detected by Summit 2.0 (CSF)	Variants Detected in Prior Tumor Genomic Profiling (*n* = 20) *Specimen Type*	Concordance
CSF-002	Lung	KRAS K117N, ARID1A Q594fs	*Brain* KRAS G12C, PBRM1 Y421_Q422delins *, *MAP2K2* amp, *PDGFRA* amp	No
CSF-004	Heme (AML)	*DNMT3A* c.1474+1G>A, DNMT3A I705T	*Bone Marrow* *ABL1* gain, *KMT2A* (11q23) rearrangement	No
CSF-005	Brain	TET2 L1065fs	*Brain* BRAF V600E, TERT c.-1446C>T, NRAS Q61K, CDK4 amp, MDM2 amp	No
CSF-001	Lung	FANCL T372fs, TP53 L257Q ^#^, *EML4::ALK* fusion	*Pleural Fluid* PDL1-negative, ALK-positive; ROS1-negative	Partial
CSF-003	Lung	EGFR L858R, DNMT3A M880V, EGFR A767V, SPEN Q2934 *, TET2 R1440fs, TP53 R110L, *CCND1* gain, *FGF3* gain, *FGF4* gain, *FGFR1* gain, *KRAS* gain, chr7p11.2 gain (*EGFR*), chr7q31 gain (*MET*)	*Pleural Fluid* *ERBB3* amp, *EGFR* amp, *FGFR1* amp, *MET *amp, *CCND1* amp, *KRAS *amp, *ERBB2* amp, *BRAF* amp, EGFR L858R, EGFR A767T *Lung* HER2-negative; MGMT not methylated, MMR normal	Yes
CSF-006	Breast	PIK3CA H1047R	*Bone Marrow* PIK3CA H1047R, chr5 gain, chr7 gain, chr8 gain, chr20q gain	Partial
CSF-007	Breast	GATA3 P409fs	*Spinous process* HER2-negative, GATA3-positive	Partial
CSF-008	Brain	H3-3A K28M, PTPN11 A72S, RB1 R358 *, RB1 Q395 *, RB1 L647fs, BCOR E1326fs, TP53 S241F ^#^	*Brain* H3F3A K28M ^#^, PTPN11 A72S, TP53 S241F, BCOR E1292fs	Partial
CSF-009	Brain	FBXW7 R465H	*Brain* SMO L412F, *TERT* c.-124C>T, FBXW7 R347H	Partial
CSF-010	Brain	DNMT3A L547fs, DNMT3A R326C	*Brain* EGFR T263P, DNMT3A L547fs	Partial
CSF-011	Lung	BRCA2 S1943fs, EGFR L858R, PPM1D W427 * chr9p21.3 loss (CDKN2A, CDKN2B, MTAP)	*Lung* BRCA2 S1943fs, EGRF L858R, EGFR L718V, *APC* loss,*CDNKA* loss, *MTAP* loss	Partial
CSF-012	Ovary	BRCA1 P798fs, ERBB4 D1104fs	*Likely Ovary* (*clinical notes not clear*) BRCA1 P798fs, POT1 T269fs, TP53 H269fs, *CCNE1* amp	Partial
CSF-013	Lung	EGFR A767_V769dup, *MYC* gain	*Likely Lung* (*clinical notes not clear*) ERGF A767_V769dup, APC L954fs, TP53 I255M, TRAF3 W420 *, *MYC* gain	Partial
CSF-014	Breast	BRCA1 E23fs, RB1 c.1696-3_1741del, RB1 c.2515_2520+47del, TP53 c.559+2T>C	*Brain* BRCA1 E23fs, *TP53* c.559+2T>C, *RB1 *c.2515_2520+47del, *RB1* c.1696-3_1741del, *AURKA* gain, *FAT1* gain, *GNAS* gain	Partial
CSF-015	Lung	*RB1* c.1961-2A>T, TP53 p.P151S, TP53 C275F, NF1 T1324A	*Endobronchial Tumor* *RB1* c.1961-2A>T, TP53 P151S	Yes
CSF-016	Lung	BRCA1 E23fs, EGFR S768_D770dup, MYD88 L265P	*Liver* EGFR S768_D770dup, BRCA1 E23fs, MYD88 L265P	Yes
CSF-017	Brain	NF1 R192 *, EGFR L62R, EGFR R108K, EGFR P596L, *PIK3R1* c.1746-2A>T, PTEN L140F, *TERT* c.-124C>T, chr7p11.2 gain (*EGFR*) *QKI::ROS1* fusion	*Brain* *EGFR*, *NF1*, *PIK3R1*, *PTEN*, and *TERT* promoter mutations, EGFR amplification, and multiple splice variants, *QKI::ROS1* fusion, *CDKN2A/CDKN2B* loss	Yes
CSF-018	Lung	ATM R1618 *, EGFR E746_S752delinsV, RAD51 R150Q, *TP53* c.559+1G>A	*Lung* EGFR E746_S752delinsV	Yes
CSF-025	Lung	EGFR E746_A750del, KDR A1065T, TP53 R213fs, ERCC2 A717G, ERCC2 L461V	*Lymph Node* EGFR E746_A750del, TP53 R213fs, *CDKN2A/CDKN2B* loss, *MTAP* loss, *TERT* gain, *TSC1* loss	Partial
CSF-028	Lung	EGFR V769_D770insGGV, TP53 R273C	*Lung* EGFR V769_D770insGGV	Yes

^#^ variant also detected in Summit 1.0; * stop-gain variant; variants listed in green are those that were detected in Summit 2.0 and known to be present in plasma and/or tumor based on clinical information provided; CSF, cerebrospinal fluid; amp, amplification.

## Data Availability

All data generated has been included in the manuscript. Any additional information can be obtained at reasonable request from the corresponding authors.

## Supplementary Materials

The following supporting information can be downloaded at: https://www.mdpi.com/article/10.3390/cancers18020256/s1, Table S1: Summit 2.0 Gene Content.
